# Turning the Knobs: The Impact of Post-translational Modifications on Carbon Metabolism

**DOI:** 10.3389/fpls.2021.781508

**Published:** 2022-01-11

**Authors:** Cleverson C. Matiolli, Rafael Cavém Soares, Hugo L. S. Alves, Isabel A. Abreu

**Affiliations:** Instituto de Tecnologia Química e Biológica António Xavier (ITQB NOVA), Universidade Nova de Lisboa, Oeiras, Portugal

**Keywords:** carbon metabolism, photosynthesis, starch, stress, PTMs, sink-source, sugar sensing

## Abstract

Plants rely on the carbon fixed by photosynthesis into sugars to grow and reproduce. However, plants often face non-ideal conditions caused by biotic and abiotic stresses. These constraints impose challenges to managing sugars, the most valuable plant asset. Hence, the precise management of sugars is crucial to avoid starvation under adverse conditions and sustain growth. This review explores the role of post-translational modifications (PTMs) in the modulation of carbon metabolism. PTMs consist of chemical modifications of proteins that change protein properties, including protein-protein interaction preferences, enzymatic activity, stability, and subcellular localization. We provide a holistic view of how PTMs tune resource distribution among different physiological processes to optimize plant fitness.

## Introduction

Plants are sessile autotrophs that thrive in ever-changing environments. These changes can be predictable, such as the daily oscillations in light and temperature or unexpected pathogen infection, flooding, and desiccation. To survive and reproduce successfully, plants must quickly adjust their metabolic activities and growth and development to overcome environmental challenges.

An essential asset of a plant’s life is the energy stored in the chemical bonds of sugars. During the day, plants capture the energy carried by the light to fix atmospheric carbon dioxide (CO_2_) into photosynthates. The fixed carbon is partitioned between sucrose, which is transported throughout the plant to feed sink tissues, and transitory leaf starch, the latter consumed during the night to sustain metabolic activities (Webb and Satake, 2015; Mathan et al., 2021). Notably, sugar and energy levels must be tightly regulated to avoid energy starvation and its detrimental effects on plant fitness.

Plants deploy extensive crosstalk between carbon metabolism and other physiological processes to coordinate growth and stress responses. Several genetic and physiological studies highlight the interaction between sugar and hormone signaling (Rolland and Sheen, 2005; Rolland et al., 2006), showing that the genetic programs organizing the production and consumption of carbohydrates are vastly complex. The balance between growth and stress tolerance is a central issue in plant fitness: plants demand carbon and energy to overcome, or at least tolerate, the environmental challenges. On the other hand, these resources are needed for growth and reproduction. More profound knowledge of the control of carbon metabolism will allow the manipulation of carbon fluxes in the plant.

Post-translational modifications (PTMs) of proteins are essential tools of the plant regulatory toolbox to regulate carbon metabolism by enabling fast, often reversible, adjustments of target protein properties. PTMs are Nature’s solution for the transduction of perceived developmental and environmental signals. They increase the proteome complexity to achieve an efficient multi-signal integration and robustness of the responses, allowing the timing of metabolism in front of parallel cues. Additionally, different PTMs can target the same protein, sometimes the same amino acid residue, or have multiple inputs in different signaling pathway components, which dramatically increases the number of proteome combinations to accommodate fine-tuned responses (reviewed in Vu et al., 2018).

The sheer complexity of the regulation of carbon acquisition and utilization by plants is quite challenging to unveil. Despite this, the precise control of metabolic carbon fluxes is an appealing strategy to improve crop yield. There are plenty of detailed reviews discussing the role of PTMs in regulating carbon metabolism in plants. They cover in detail most of the aspects of carbon and energy-sensing (Ramon et al., 2008; Baena-González and Hanson, 2017; Margalha et al., 2019), carbon fixation (Houtz et al., 2008; Grabsztunowicz et al., 2017), starch metabolism (Kötting et al., 2010; Abt and Zeeman, 2020), and general effects of PTMs on plant metabolism (Friso and Van Wijk, 2015). However, discoveries in these research fields are frequent because a large community is engaged in dissecting carbon pathways in plants. Thus, in the present review, we provide a holistic update on the effects of PTMs in carbon metabolism. We discuss relevant updates on the carbon flow from the fixation of atmospheric CO_2_ into sugars to hexose breakdown by glycolysis. We sought to cover some critical aspects of this extensive issue in plant physiology by approaching the role of PTMs in carbon metabolism: sensing; fixation; storage and remobilization; transport, and cytosolic glycolysis.

## Sensing Carbon and Energy: The Eukaryotic Conserved Masters of Carbon Flux Control

**FIGURE 1:**
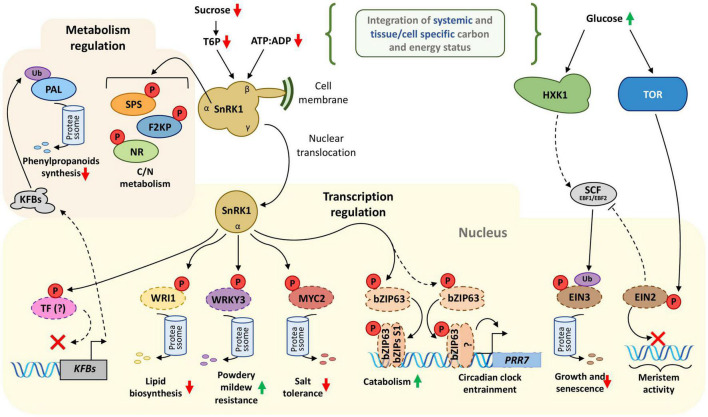
The evolutionary conserved carbon and energy-sensing in plants. Sugars and energy levels are detected by SnRK1 and TOR sensors, which in turn phosphorylate enzymes and transcription factors (TFs) to shift the balance between anabolic and catabolic processes to attain metabolic homeostasis. The hexose-phosphorylating enzyme HXK1 has catalysis-independent sugar sensing activity, which regulates ethylene signaling and the gene expression of photosynthesis-related genes. Sugar levels modulate SnRK1 activity (see the article for details on the sucrose-T6P nexus regulating SnRK1 activity) and ATP:ADP ratio. SnRK1 phosphorylates enzymes involved in carbon and nitrogen metabolism – such as SPS, F2KP, and NR – to coordinate the metabolic demands of growth and stress responses. SnRK1 phosphorylates and destabilizes several TFs – namely WRI1, WRKY3, and MYC2 – to adjust the physiological responses to a plethora of internal and external cues (e.g., carbon and energy starvation; biotic and abiotic stresses). The phosphorylation of the sugar-responsive circadian clock regulator bZIP63 by SnRK1 modifies its dimerization pattern with C/S1-bZIPs group, activating the transcription of genes associated with catabolism to supply metabolic demands. Additionally, bZIP63 entrains the circadian clock in response to sugars by inducing *PRR7* transcription, possibly relying on SnRK1 signaling. SnRK1 likely targets a still unidentified TF to modulate the expression of specific KFBs, which are E3-ubiquitin ligases containing Kelch motifs. These KFBs ubiquitinate and target the PAL enzyme to degradation, decreasing the phenylpropanoids biosynthesis. EIN3, an ethylene-responsive TF, appears to be at the interface of the molecular responses to both energy deficit and availability. EIN3 stability is putatively modulated by SnRK1 and HXK1, which respond to antagonistic signals: dropping or rising sugar levels, respectively. SnRK1 directly phosphorylates EIN3 to destabilize it. Glucose availability perceived by HXK1 indirectly triggers EIN3 degradation, a process that could involve SCF*^EBF1/2^*-mediated EIN3 ubiquitination. TOR prevents the nuclear translocation of EIN2, which in turn inhibits EBF1/2 translation and thus EIN3 degradation, highlighting a potential role of EINs in the crosstalk between hormonal and energy/nutrient signaling. Interestingly, phosphorylation and ubiquitination seem to be fundamental PTMs that translate the energy and stress signals perceived by SnRK1, HXK1, and TOR. Ellipses and other rounded shapes = proteins (dashed lines: transcription factors (TFs), solid lines: enzymes). Phosphorylation (P); ubiquitination (Ub). Connectors ending with arrows = activation; connectors ending with bars = repression. Solid connectors = demonstrated pathways; dashed connectors = hypothetic pathways. Green upward arrows = induction; red downward arrows = repression.

The utilization of carbon and energy is tightly regulated to avoid the detrimental effects of starvation (Moraes et al., 2019; Viana et al., 2021). The adequate management of sugar status, which represents the amount of carbon and energy readily available in the system, requires cellular mechanisms to detect sugar levels. These sensing mechanisms are integrated into stress-responsive regulatory networks, allowing crosstalk between developmental and environmental signals to coordinate plant metabolism and growth. Additionally, monitoring the sugar availability in different plant parts is key to determining the (re)distribution of sugars throughout the organism. In the last two decades, the role of three eukaryotic conserved kinases in maintaining carbon-energy-nutrient homeostasis has become apparent. These are the SUCROSE NON-FERMENTING RELATED KINASE1 (SnRK1) and TARGET OF RAPAMYCIN (TOR), which are pivotal protein kinases regulating carbon and nitrogen metabolism, and the hexose-phosphorylation enzyme HEXOKINASE1 (HXK1). The concerted action of these three kinases promotes carbon-energy-nutrient homeostasis, which is crucial to ensure plant fitness in changing environments (Figure 1).

The energy-sensing kinase SnRK1 is a central hub integrating stress responses with carbon and energy metabolism in plants (Figure 1; Wurzinger et al., 2018; Margalha et al., 2019; Alves et al., 2021). Dropping sugar and energy levels activates SnRK1, which in turn phosphorylates enzymes and transcription factors to reprogram the metabolism (Baena-González et al., 2007; Mair et al., 2015; Nietzsche et al., 2016). Rising SnRK1 activity favors catabolism rather than anabolism, thus repressing growth and activating energy recovery from alternative carbon sources (Baena-González et al., 2007; Nukarinen et al., 2016; Pedrotti et al., 2018). For instance, SnRK1 phosphorylates and inactivates SUCROSE PHOSPHATE SYNTHASE (SPS), NITRATE REDUCTASE (NR), and FRUCTOSE-2,6-BIPHOSPHATASE (F2KP) (Figure 1) [overviewed in Nägele and Weckwerth (2014)]. The inactivation of these enzymes by SnRK1 aims to redirect carbon from anabolism, such as sucrose synthesis and export, to catabolic energy-generating pathways. Additionally, the regulation of NR activity by SnRK1 is a possible point of crosstalk for carbon and nitrogen metabolism.

A great deal of SnRK1 complex regulation is that it accommodates multiple environmental and metabolic signals. The complex regulation is thought to be achieved through an array of possible configurations of SnRK1 complex subunits in addition to upstream PTMs or allosteric regulations. Briefly, SnRK1 is a trimeric protein complex containing one catalytic (α-subunit) and two regulatory β- and γ-subunits (Crepin and Rolland, 2019). However, how the subunits interact to manage the diverse set of signal inputs received by the trimeric complex is still poorly understood. SnRK1 is inhibited by trehalose-6-phosphate (T6P), which communicates the sucrose levels in the plant (sucrose-T6P nexus; reviewed in Baena-González and Lunn, 2020). Interestingly, a recent report demonstrates that the SnRK1 α-subunit alone shows independent catalytic activity when dissociated from its regulatory subunits. Upon energy limitation, i.e., a drop in sugar levels and ATP:ADP ratio, the catalytic SnRK1α subunit dissociates from the membrane-bound β-subunits. After the dissociation from the β-subunit, the SnRK1 catalytic α-subunit translocates to the nucleus to regulate gene expression through the phosphorylation of low-energy responsive transcription factors (TFs) (Figure 1; Ramon et al., 2019). The transcriptional reprogramming triggered by SnRK1 is partially mediated by the C/S1-group of bZIP transcription factors (Baena-González et al., 2007; Matiolli et al., 2011; Viana et al., 2021). The phosphorylation of bZIP63 by SnRK1 is followed by rearranging the heterodimerization of bZIP63 with the C/S1-group of bZIPs (Mair et al., 2015). The resulting bZIP63-S1 heterodimers promote the expression of catabolism-associated genes to recover energy from alternative sources, such as amino acids, to sustain respiration and enhance survival. Additionally, both SnRK1 and bZIP63 mediate the entrainment of the Arabidopsis circadian oscillator by sugars (Frank et al., 2018), which is thought to synchronize plant metabolism to daily external cues (Figure 1). The influence of SnRK1 and bZIP63 on the circadian oscillator might be essential to modulate starch degradation and plant growth (Viana et al., 2021).

SnRK1 also modulates the activity of other transcription factors regulating genes associated with carbon and energy metabolism. For instance, SnRK1 phosphorylates the AP2-type TF WRINKLED1 (WRI1) to trigger its degradation by the 26S proteasome, which in turn represses lipid biosynthesis when the cellular sugar level is low (Zhai et al., 2017). SnRK1 phosphorylation also triggers the degradation of the basic helix-loop-helix (bHLH) MYC2 to antagonize salt tolerance (Im et al., 2014). In barley, SnRK1 phosphorylation destabilizes WRKY3 to enhance the resistance to powdery mildew (Han et al., 2020), possibly modulating carbon homeostasis to optimize growth-defense balance (Figure 1). Phenylpropanoid synthesis is a strong sink for fixed carbon in plants, synthesizing lignin and secondary metabolites for defense. When activated by energy starvation, SnRK1 represses the accumulation of phenylpropanoids by downregulating the transcription of a set of KELCH DOMAIN-CONTAINING F-BOX (KFB) proteins. These KFB proteins ubiquitinate the PHENYLALANINE AMMONIA-LYASE (PAL) and assign it for degradation (Figure 1). The transcription of KFB genes by SnRK1 could be mediated by SnRK1-dependent phosphorylation of still unidentified transcription factors (Wang et al., 2021). The evidence suggests a clear relationship between phosphorylation and ubiquitination, two of the most common PTMs in plants, prompting us to uncover how these two PTMs crosstalk to integrate different signals.

Efficient environmental and metabolic signal integration requires multi-level interaction between different signaling pathways. For instance, SnRK1 shares targets, including carbon and nitrogen metabolic enzymes, with several calcium-dependent protein kinases (CDPKs) (reviewed in Alves et al., 2021). Interestingly, SnRK1 and HXK1 signaling pathways converge in the regulation of the ETHYLENE INSENSITIVE3 (EIN3) transcription factor protein stability (Moore et al., 2003; Yanagisawa et al., 2003; Kim et al., 2017). Ethylene is a gaseous hormone that regulates growth and senescence, two critical physiological processes inextricably bound to the carbon and energy economy. While glucose availability induces EIN3 degradation mediated by HXK1 glucose sensing-activity, SnRK1 directly phosphorylates EIN3 to enable its degradation (Figure 1). The proteolysis of EIN3 triggered by HXK1-mediated glucose sensing and SnRK1 direct phosphorylation is likely mediated by the SCF^EBF1/EBF2^ complexes containing the F-box proteins EIN3-binding F-box1 or 2 (EBF1/EBF2) that ubiquitinate the transcription factor EIN3 (Gagne et al., 2004). Phosphorylation and degradation of EIN3 slow down leaf senescence, suggesting a fine-tunning mechanism to regulate developmental aging of photosynthetic tissues according to carbon status. Interestingly, phosphorylation of ETHYLENE INSENSITIVE2 (EIN2) by TOR prevents its nuclear localization (Fu et al., 2021) and partially mediates the glucose-activated gene expression reprogramming orchestrated by TOR to regulate meristem activity (Xiong et al., 2013). The putative increase of the EIN2 pool outside the nucleus might enhance its role as a repressor of EBF1/2 translation (Li et al., 2015), thus inhibiting EIN3 degradation. The crosstalk between SnRK1, TOR, and HXK1 to modulate ethylene signaling in response to carbon status is a clear example of the multi-level regulation of critical developmental signaling pathways by PTMs.

TOR is responsible for monitoring resource availability to activate metabolic processes associated with biosynthesis and growth. Thus, TOR and SnRK1 have antagonistic effects on plant energy and carbon metabolism. While SnRK1 is activated by energy and carbon deprivation, TOR is activated by carbon and nutrient availability to promote translation and meristem activation in response to sugars and amino acids (Xiong et al., 2013; Cao P. et al., 2019; Liu et al., 2021). In plants, TOR acts in a protein complex composed of two more regulatory proteins: RAPTOR1B and LST8 (Dobrenel et al., 2016). The SnRK1 α-subunit interacts with RAPTOR1B in the cytoplasm of plant cells and phosphorylates RAPTOR1B *in vitro* (Nukarinen et al., 2016), resembling the regulatory crosstalk observed in yeast and mammals for the ortholog counterparts of the plant SnRK1 and RAPTOR1B. The regulatory crosstalk between SnRK1 and TOR pathways is thought to balance anabolism and catabolism to maintain carbon and nutrient homeostasis. For instance, TOR inhibition disrupts the carbon/nitrogen (C/N) balance under carbon limitation in *Chlamydomonas reinhardtii* (Mubeen et al., 2019).

The regulator of G-protein signaling1 (RGS1) is a transmembrane protein localized in the plasma membrane that senses D-glucose (Chen and Jones, 2004), coordinating cell proliferation and hypocotyl elongation in response to glucose (Chen et al., 2003). There are fewer studies analyzing the function of RGS1 in sugar sensing and carbon metabolism regulation, but recent evidence links RGS1 to plant immunity. Plant immunity can be a taxing physiological process in plants. On one side, pathogens try to hijack carbon, energy, and nutrients from the host plant. On the other side, the plant deploys both evasive and attacking strategies to fend off the invaders. The primary response to biotic stress relies on detecting microbe-associated molecular patterns (MAMPs) by specific cell membrane receptors. Liang et al. (2018) showed that RGS1 maintains the flagellin receptor FLAGELLIN-SENSITIVE2 (FLS2) in an inactive form by forming a protein complex (Liang et al., 2018). Upon detecting bacterial flagellin or its conserved peptide flg22, RGS1 is phosphorylated by FLS2-bound kinase BRASSINOSTEROID INSENSITIVE 1-associated receptor kinase 1 (BAK1) to promote the release of FLS2 and subsequent downstream activation of cytoplasmic kinases. One can hypothesize that this mechanism may act as a rheostat balancing glucose availability with the costly defense activation.

## Carbon Fixation: Photosynthesis

**FIGURE 2:**
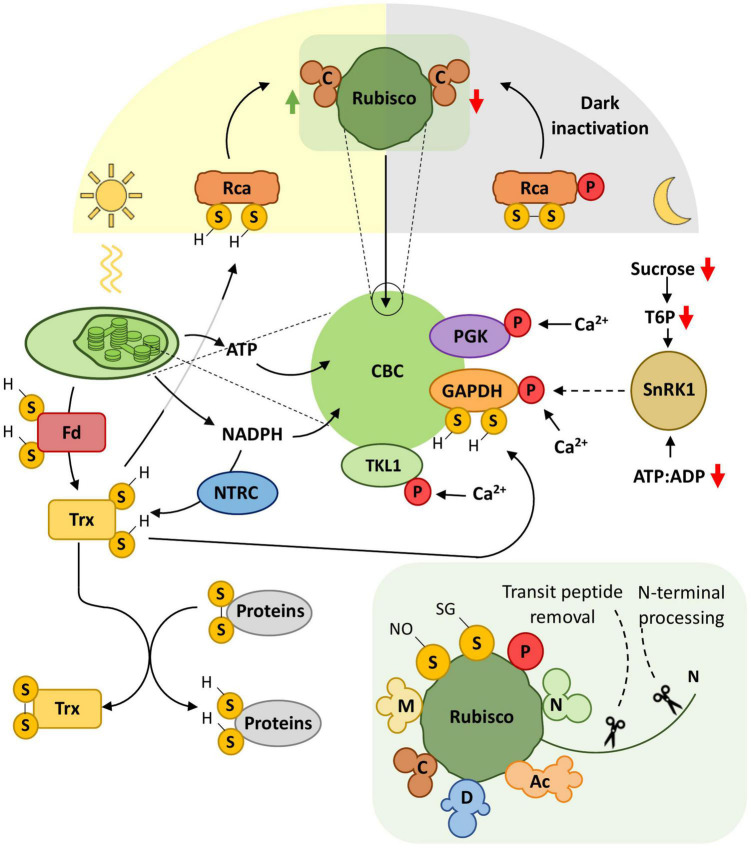
PTMs modulate carbon fixation. The carbon assimilation in plants is heavily regulated to allow its adjustment to a plethora of incoming signals from metabolism and the external environment. The figure depicts the photosynthetic carbon fixation through the Calvin-Benson cycle (CBC) and Rubisco activity, coupled to the Fd/Trx and NTRC complexes and ATP formation. The major signals modulating the CBC activity are light/dark transitions, redox status, sugar availability, Ca^2+^ levels, and ATP:ADP ratio. Light powers photosynthesis to generate energy (ATP) and reducing power in the form of NADPH. NTRC uses NADPH as a substrate to reduce Trx, in a mechanism not necessarily dependent on light. Importantly, NADPH allows the communication between the Fd/Trx and NTRC complexes. Trx sulfhydryl groups reduce several proteins, including CBC enzymes, to regulate their activity. One crucial example is Rca, whose reduction state directly affects Rubisco activity. During the day, reduced Rca removes inhibitory RuBP and facilitates Rubisco carbamylation to enhance its carboxylase activity. During the night, oxidized and phosphorylated Rca cannot reactivate Rubisco, shutting down photosynthesis. The activity of other CBC enzymes, such as PGK, TKL1, and GAPDH, is modulated via Ca^2+^-dependent phosphorylation, which highlights the integration of developmental signals and environmental stresses into photosynthesis regulation. GAPDH is a putative *in vivo* direct target of SnRK1 and is also modulated by the redox state through Trx. The SnRK1-Trx regulation of GAPDH activity may act as a central hub in the CBC to adjust carbon and energy metabolic fluxes accordingly to tissue-specific and/or developmental demands. Inset figure: Rubisco is regulated by a myriad of PTMs, highlighting its central role in carbon fixation. Phosphorylation (P), tyrosine nitration (N), lysine methylation (M), acetylation (Ac), sulfhydryl groups (S-H), disulfide bridge (S-S), nitrosylation (S-NO), glutathionylation (S-SG), deformylation (D), carbamylation (C). Connectors ending with arrows = activation; connectors ending with bars = repression. Solid connectors = demonstrated pathways; dashed connectors = hypothetic pathways. Green upward arrows = induction; red downward arrows = repression.

Chloroplasts are power generators of plant cells: they convert the energy carried by light into the chemical energy of covalent bonds of sugars. These sugars can be stored as semi-crystalline starch granules, the “battery” keeping the plant metabolism running in the absence of light or transported from photosynthetic tissues (source) in the form of sucrose to non-photosynthetic or young tissues (sink). The light energy is captured by the light-harvesting complex (LHC) to produce reducing power in the form of NADPH and create a proton gradient between the thylakoid lumen and the chloroplast stroma. The proton gradient is then converted into ATP by the ATP-synthase embedded in the thylakoid membrane. The NADPH and ATP are used in the series of carbon-fixing reactions inside the chloroplast. The light input also triggers redox signaling by allowing the reduction of disulfide bridges in chloroplastic proteins through the ferredoxin/thioredoxin (Fd/Trx) system and NADP-dependent thioredoxin reductase C (NTRC) (Nikkanen and Rintamäki, 2019). The reducing power stored in the thioredoxins is used to reduce enzymes of the Calvin-Benson cycle (CBC) (reviewed in Selinski and Scheibe, 2019). Notably, the Fd/Trx and NTRC-mediated reduction-signaling pathways complement each other during light conditions, and they also can communicate among them. The Fd/Trx system affects NTRC substrate availability by balancing the chloroplastic NADPH/ATP ratio through ferredoxin-NADP reductase and the reduction-activation of NADP-malate dehydrogenase (NADP-MDH) (Ceccarelli et al., 2004; Lemaire et al., 2005; Yokochi et al., 2021). Thioredoxin also affects ATP synthesis directly through the reduction-activation of chloroplast ATP synthase (Schumann et al., 1985). NADP-MDH synthesizes malate from oxaloacetate, which is easily transported and readily used as an indirect source of reducing equivalents or for ATP synthesis. The regulation of the redox state of enzymes associated with carbon fixation and starch metabolism is one of the most relevant PTMs modulating carbon metabolism (Figure 2).

Photosynthetic carbon fixation starts when atmospheric CO_2_ is assimilated into 3-phosphoglycerate (3PG) in a reaction catalyzed by Ribulose-1,5-bisphosphate (RuBP) carboxylase/oxygenase (Rubisco). As a central enzyme in carbon fixation, Rubisco is heavily regulated by various PTMs, namely: phosphorylation, tyrosine nitration, acetylation, lysine methylation, nitrosylation and glutathionylation, N-terminal processing, deformylation, transit peptide removal, and carbamylation (reviewed in Grabsztunowicz et al., 2017; Figure 2). The reactivation of Rubisco by the carbamylation of the lysine in the Rubisco active site relies on the regulation of the chaperone Rubisco activase (Rca) by both the redox state and ATP/ADP ratio in the chloroplast (Carmo-Silva et al., 2015). The Fd/Trx system reduces Rca disulfide bridges, resulting in increased Rca activity (Zhang and Portis, 1999). Activated Rca continuously remodels inhibited active sites of Rubisco by removing inhibitory RuBP to enhance photosynthetic activity (Carmo-Silva et al., 2015; Perdomo et al., 2021; Figure 2). Moreover, Rca dark-dependent inactivation is putatively regulated by Thr78 and Ser172 phosphorylation (Boex-Fontvieille et al., 2014). Rca phosphorylation relocates it to the thylakoid membrane, where Rca protects the chloroplast Serine/threonine-protein kinase (Stt7) from proteolysis. Stt7 participates in balancing excitation energy between photosystem I and II for photosynthetic yield optimization (Lemeille et al., 2010). The phosphoproteome of the *Chlamydomonas* mutant *stt7* revealed three groups of thylakoid membrane proteins regulated by phosphorylation: (i) Stt7-dependent (composed mostly of LHCII proteins); (ii) redox-dependent (independently of Stt7); and (iii) redox-independent (Lemeille et al., 2010).

Besides Rubisco, other CBC enzymes are regulated by phosphorylation. PHOSPHOGLYCERATE KINASE (PGK), GLYCERALDEHYDE 3-PHOSPHATE DEHYDROGENASE (GAPDH), and TRANSKELOTASE1 (TKL1) are phosphorylated in a Ca^2+^-dependent manner (Reiland et al., 2009; Piattoni et al., 2017), suggesting that the activity of these enzymes is modulated by internal and external cues triggering cellular [Ca^2+^] changes, such as developmental signals and environmental stresses. Interestingly, GAPDH is phosphorylated by the SnRK1 *in vitro*, and its activation mechanism also requires redox regulation (Holtgrefe et al., 2008; Piattoni et al., 2017; Schneider et al., 2018; Figure 2). A sugar-derived signal might affect this process because tissues with different carbon reserves present different SnRK1 activities and phospho-GAPDH profiles (Piattoni et al., 2017). GAPDH seems to act as a central hub for regulating energy supply and balancing carbon and energy metabolic fluxes (Schneider et al., 2018).

## Carbon Storage and Remobilization: Starch Metabolism and Autophagy

A share of the photoassimilates is stored as starch during the day. The partitioning of photoassimilates is heavily regulated to accommodate current cellular metabolic activities and storage for later use and the likely occurrence of environmental fluctuations in light and carbon utilization demand to fight stress. For instance, transitory starch synthesis and degradation can coincide during long-day or low-light end-of-day conditions (Fernandez et al., 2017) or during stress (Thalmann and Santelia, 2017). Various enzymes control starch synthesis and degradation, and some take part in both processes (reviewed in Kötting et al., 2010). Additionally, several starch metabolic enzymes have differential spatio-temporal expression patterns (Qu et al., 2018). All these factors point to a complex and concerted regulation of starch enzymes’ activity with temporal and spatial parameters.

The major transitory starch synthesis pathway starts with the conversion of glucose-6-phosphate (G6P) to glucose-1-phosphate (G1P) by the plastidial protein PHOSPHOGLUCOMUTASE1 (PGM1), an enzyme that catalyzes the interconversion G6P ↔ G1P (Caspar et al., 1985). While the conversion of G6P to G1P is an essential step of starch synthesis, the generation of G6P from G1P can feed glucose to glycolysis or to anabolic reactions during starch degradation. Arabidopsis mutants lacking a functional PGM1 enzyme accumulate around 1% of the starch of a wild type plant, severely impairing C-net fixation throughout the diel cycle and growth (Usadel et al., 2008). PGM1 is S-nitrosylated in multiple sites (Hu et al., 2015), leading to the hypothesis that PGM1 activity could be modulated by nitric oxide (NO) levels. Interestingly, high NO levels inhibit starch accumulation in Arabidopsis (Zhang et al., 2017). It could be interesting to investigate if PGM1 S-nitrosylation plays a role in the regulation of starch synthesis. Additionally, a quantitative phosphoproteomic analysis of the Arabidopsis ABA-insensitive triple mutant *snrk2.2/2.3/2.6* revealed that PGM1 could be phosphorylated in response to ABA (Wang et al., 2013), resulting in the putative modulation of photosynthetic carbon flow into or from starch in response to abiotic stress.

**FIGURE 3:**
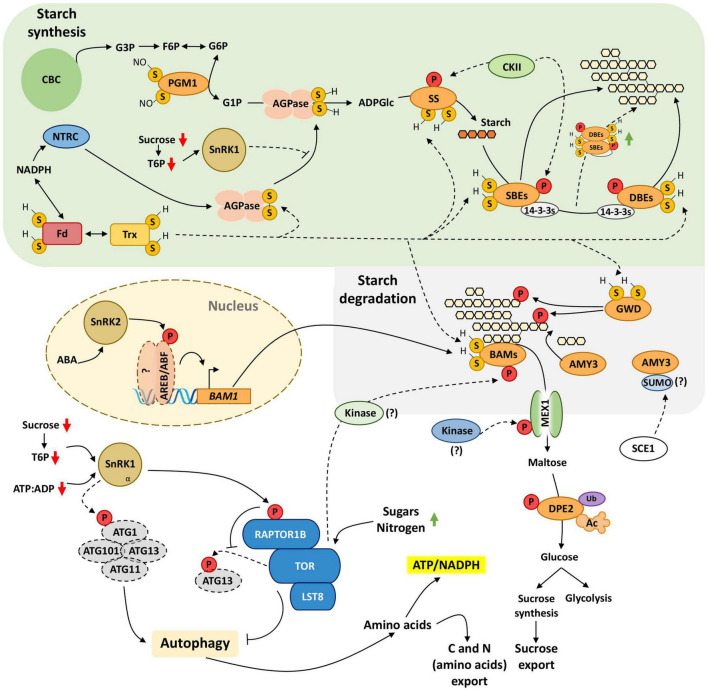
Cellular redox status and phosphorylation control critical steps of starch metabolism. PTMs serve for tight, transient control of enzymes involved in starch metabolism to meet the energy demands associated with daily growth and stress responses. NTRC uses the NADPH to reduce and activate AGPase. Trx can also reduce AGPase *in vitro*, but the *in vivo* evidence is still lacking. Here, we hypothesize that Trx could also regulate the redox state of other enzymes associated with starch metabolism. AGPase might also integrate systemic and tissue-specific signals conveying the carbon status, putatively through the sucrose-T6P nexus and SnRK1 regulatory axis. AGPase synthesizes the substrate of starch polymerization, ADP-Glucose (ADP-Glc), which enters the starch synthesis pathway performed by a set of enzymes that elongates and control the branching of starch polymer (e.g., SS, SBEs, and DBEs). The phosphorylation of these enzymes seems to generally enhance their activity, possibly through the formation of metabolic enzyme complexes. The binding of 14-3-3 proteins to SBEs and DBEs might also promote enzyme complex formation to increase starch polymerization efficiency. Redox regulation is also necessary for starch degradation as the reduced GWD phosphorylates starch to open its structure and increase starch availability to amylases. Beta-amylases (BAMs) catalyze starch breakdown into maltose, and this crucial step might integrate hormonal (ABA) and nutrient signals through SnRK2-AREB/ABF pathway and TOR. While SnRK2 enhances *BAM1* expression, TOR-dependent BAM1 phosphorylation might enhance its activity. The physiological relevance of several PTMs of proteins involved in starch degradation, such as alpha-amylase AMY3, maltose export from chloroplast (MEX1), and conversion to glucose (DPE2), is yet to be clarified. Nonetheless, protein disulfide reduction and phosphorylation seem to be essential PTMs regulating starch synthesis/degradation dynamics. Upon C-starvation, SnRK1 promotes autophagy processes through ATG1 phosphorylation, activating the ATG1 kinase complex (ATG1, AGT13, ATG101, and ATG11). SnRK1 also phosphorylates RAPTOR1B and disrupts the TOR kinase complex that inhibits the ATG1 complex. The SnRK1-TOR axis regulates starch degradation and autophagy to promote energy and nutritional homeostasis at the cellular and systemic levels. Phosphorylation (P), ubiquitination (Ub), acetylation (Ac), sulfhydryl groups (S-H), disulfide bridge (S-S), nitrosylation (S-NO). Connectors ending with arrows = activation; connectors ending with bars = repression. Solid connectors = demonstrated pathways; dashed connectors = hypothetic pathways. Green upward arrows = induction; red downward arrows = repression. Starch is represented by many linked glucose moieties, while fewer linked glucose moieties represent starch degradation products.

Redox-regulation and phosphorylation are the prevailing PTMs modulating starch metabolism (Figure 3). ADP-glucose (ADPGlc) synthesis by ADP-glucose pyrophosphorylase (AGPase) is a key regulatory node for directing carbon to starch granule formation according to the plastid redox state (Michalska et al., 2009). The AGPase is a heterotetrameric holoenzyme composed of two small catalytic (APS) and two large (APL) subunits. They are arranged in two pairs APS-APL heterodimers linked by a disulfide-bridge between the two APS subunits (Geigenberger et al., 2005; Hädrich et al., 2012). The AGPase connects photosynthesis to starch metabolism through the Fd/Trx system and the NADP-dependent thioredoxin reductase C (NTRC) (Hendriks et al., 2003; Skryhan et al., 2018). The AGPase heterotetramer is activated *in vitro* by thioredoxin *f/m* and NTRC when the disulfide bridge formed by the APS subunits’ Cys82 residues is reduced (Michalska et al., 2009; Geigenberger, 2011). NTRC has also been shown to modulate AGPase activity *in vivo* and regulate the AGPase independently of light via NADPH generated by sugar catabolism (Michalska et al., 2009). The AGPase redox state, and thus starch synthesis, is also modulated by SnRK1 (Figure 3). Redox activation of other starch biosynthetic enzymes was also observed *in vitro* for starch synthases (SS), branching (SBE), and debranching enzymes (DBE), such as the Arabidopsis SS1, SS3, SBE2, and the spinach pullulanase (Schindler et al., 2001; Glaring et al., 2012). Overexpression of SnRK1 in Arabidopsis decreases the amount of AGPase in the active reduced state (Geigenberger et al., 2005; Jossier et al., 2009). Interestingly, the artificial increase of T6P through the overexpression of TREHALOSE PHOSPHATE SYNTHASE1 (TPS1) also leads to the accumulation of AGPase in a monomeric state (Kolbe et al., 2005). The increase of AGPase in the monomeric state could be reproduced by sucrose but not glucose, suggesting that the process may obey the sucrose-T6P nexus (Baena-González and Lunn, 2020; Peixoto et al., 2021). The increase in sucrose levels leads to increased T6P accumulation, a process that might be regulated by SnRK1 (Peixoto et al., 2021), which in turn, feedback regulates carbon partitioning between sucrose and starch. Further analysis considering the diel fluctuation in the sink and source tissues on the regulation of SnRK1 and T6P in starch metabolism is needed to clarify this pathway.

Starch granule formation is catalyzed by SS, SBE, and debranching enzymes (DBE), as depicted in Figure 3. To initiate the synthesis of insoluble glucan composed of amylose and amylopectin polymers, SSs transfer the glucosyl-moiety of ADPGlc to the acceptor molecule α-1,4-D-glucan, performing α-1,4 linkage (glucan elongation). SBEs perform transglycosylation of α-1,4 linkages to α-1,6 branch points between the same or different α-1,4-D-glucans molecules (glucan branching). DBEs, which cleave α-1,6- linkages, are thought to curate the glucan branching points to allow correct granule formation. Increasing evidence supports the regulation of starch granule formation-associated enzymes through phosphorylation. The Arabidopsis SS2 is phosphorylated at the end of the dark period, at the region S63/65, which holds the canonical binding motif of Casein Kinase II (CKII) (Reiland et al., 2009; Patterson et al., 2018). Although the functional relevance of this phosphorylation is unclear, chloroplastic CKII also phosphorylates other starch biosynthetic enzymes such as fibrillin and SBE2.1 *in vitro* (Schönberg et al., 2014). Fibrillin interacts with SS4, allowing the complex to associate with specific thylakoid regions where starch granules formation may initiate (Gámez-Arjona et al., 2014; Schönberg et al., 2014). Phosphorylation also affects complex formation between SSs, SBEs, and other proteins, which may increase starch polymerization efficiency (Tetlow et al., 2004, 2008; Liu et al., 2012; Mehrpouyan et al., 2021). Barley SS and SBEs have been found to interact with 14-3-3 proteins, which can form dimers and bind to phosphorylated client proteins (Figure 3; Alexander and Morris, 2006). Thus, 14-3-3 proteins may act as a scaffold for the formation of starch metabolic enzyme complexes. In wheat, phosphorylation activates SBEIIa and SBEIIb in chloroplasts and amyloplasts, respectively, while dephosphorylation decreases their activities (Tetlow et al., 2004).

The GLUCAN WATER DIKINASE (GWD) phosphorylates the C6 position of glucose moieties in starch granules, opening its structure and consequently increasing the accessibility of β-amylases (BAMs) to the polymer (Figure 3), allowing efficient starch degradation (Reimann et al., 2004; Ritte et al., 2006; Edner et al., 2007). The redox state of GWD strongly affects its activity *in vitro*, being rendered almost totally inactive after being oxidized while its reduction could revert this effect (Mikkelsen et al., 2005). The major product of leaf starch breakdown is maltose, a reaction catalyzed by BAMs (Weise et al., 2005). In Arabidopsis, the degradation of starch into maltose is catalyzed by β-AMYLASE1 (BAM1) and β-AMYLASE3 (BAM3), a process in which the latter plays a major role in transitory leaf starch degradation. Knockout *bam3* mutant plants show stunted growth and strong starch-excess phenotype, while *bam1* has slightly higher starch accumulation at the end of the night compared to wild type plants. Importantly, knocking out BAM3 exacerbates the latter phenotype (Caspar et al., 1991; Fulton et al., 2008), suggesting that they operate cooperatively to convert starch into maltose. The redox-regulated BAM1 is the only BAM enzyme activated in reducing conditions *in vitro*, especially by thioredoxin *f* and NTRC (Sparla et al., 2006; Valerio et al., 2011). Interestingly, BAM1, but not BAM3, is associated with a diurnal starch breakdown in osmotically stressed mesophyll and guard cells for osmolytes production to open the stomata in the morning (Valerio et al., 2011). The N-terminal of BAM1 (Ser31) is phosphorylated in a TOR-dependent manner (Figure 3; Van Leene et al., 2019). How this phosphorylation influences BAM1 activity is still unknown, but it is reasonable to hypothesize that the TOR-mediated phosphorylation could activate BAM1 to remobilize carbon from starch to growth and to open the stomata.

BAM1 and α-AMYLASE3 (AMY3) act synergistically to promote stomatal opening in the light and under osmotic stress. The AREB/ABF-SnRK2 kinase-signaling pathway increases the activity of BAM1 in response to ABA treatment by enhancing BAM1 transcription (Thalmann et al., 2016). In the same work, the authors showed that the *amy3 bam1* double mutant has impaired root growth, suggesting that this ABA-regulatory pathway adjusts carbon utilization from starch to meet both growth and stress tolerance demands. AMY3 was found to interact with SCE1 in a large-scale screening for proteins interacting with SUMOylation machinery (Elrouby and Coupland, 2010), suggesting that AMY3 might be regulated by SUMOylation (Figure 3). The degradation of starch by BAMs releases maltose that is transported to the cytosol through the maltose transporter MALTOSE EXCESS1 (MEX1) (Niittylä et al., 2004). MEX1 localized in the chloroplast envelope and was found to be phosphorylated at Ser76 in a large-scale phosphoproteomic assay (Figure 3; Nakagami et al., 2010). It could be interesting to investigate if the phosphorylation of Ser76 regulates MEX1 transport activity in response to the cellular carbon status. The maltose exported from the chloroplast is processed by the maltotriose-metabolizing enzyme DISPROPORTIONATING ENZYME2 (DPE2) to generate glucose for glycolysis and sucrose synthesis for export during the night. A quick search for Arabidopsis DPE2 PTMs using the PTM-Viewer webtool^1^ revealed that DPE2 undergoes multiple modifications, including acetylation, ubiquitination, and phosphorylation (Figure 3). Further investigation of these PTMs, as well as its effectors, may shed light on the regulation of carbon flux from the chloroplast to the cytosol for glycolysis and sucrose synthesis.

Autophagy is an evolutionary conserved catabolic process encompassing both selective and non-selective degradation of cytosolic material. It recycles carbon, nitrogen, and energy to sustain respiration, growth, and reproduction. In plants, the attack of condemned proteins and organelles by lytic enzymes takes place in vacuolar autophagic bodies that are originated either by the engulfment of cytosolic material through tonoplast invagination (i.e., microautophagy) or by the vacuolar fusion with the autophagosome carrying proteins, organelles, and large portions of the cytosol (i.e., macroautophagy, hereafter autophagy) (Li and Vierstra, 2012). Autophagy plays a vital role in plants by keeping adequate energy and nutrient levels throughout development, particularly upon exposure to adverse environmental conditions (Thompson et al., 2005; Izumi et al., 2013; Michaeli et al., 2016). The AUTOPHAGY RELATED1 (ATG1) kinase complex is composed of four subunits (ATG1/ATG13/ATG11/ATG101) (Figure 3) and initiates the autophagic process by phosphorylating proteins and targeting them for destruction (Kijanska and Peter, 2013). Nutrient deficiency and environmental stresses activate SnRK1, which promotes autophagy to remobilize resources needed to sustain respiration and cope with stress. Part of this regulation is thought to be achieved through the repression of the TOR complex. SnRK1 catalytic α-subunit interacts with the TOR complex regulatory subunit RAPTOR1B and phosphorylates it *in vitro* (Nukarinen et al., 2016). On the other hand, ATG13, a member of the ATG1 kinase complex, is phosphorylated in multiple sites in a TOR-dependent manner (Van Leene et al., 2019). Thus, RAPTOR1B phosphorylation by SnRK1 is likely to alleviate the repression of the ATG1 kinase complex exerted by the TOR complex activity (Figure 3). SnRK1 can also regulate autophagy through direct or indirect phosphorylation of ATG1 protein in the ATG1 kinase complex (Chen et al., 2017). Recently, Huang et al. (2019) proposed that SnRK1 regulates autophagy through two distinct pathways: short-term C-starvation triggers the phosphorylation of ATG1 protein, while long-term C-starvation signal is conveyed by ATG1-independent signaling pathway to activate the ATG1 kinase complex (Huang et al., 2019).

Autophagy and starch degradation seem to act synergistically during the night to optimize growth because double mutants impaired in both processes have enhanced dwarf phenotype compared to the single mutants (Izumi et al., 2013; Figure 3). SnRK1 may act as a hub coordinating starch degradation and autophagy to supply energy and nutrients required by growth and stress responses. We discussed before that TOR inhibition disrupts the carbon/nitrogen (C/N) balance under carbon limitation caused by extended darkness in *Chlamydomonas reinhardtii* (Mubeen et al., 2019). Moreover, extended darkness leads to carbon starvation due to the exhaustion of the transitory starch accumulated for the duration of the night. Thus, the additive effect of starch degradation and autophagy on growth likely involves the coordination of SnRK1 and TOR pathways, ensuring the availability of energy and amino acids for translation. Altogether, the SnRK1-TOR regulatory axis dynamically modulates starch and autophagy remobilization to optimize resource utilization under nutrient and carbon-energy stress.

As we see, recycling carbon and energy is essential in stress responses and plant development. It is becoming clear that ubiquitination, and its interaction with phosphorylation, plays a large role in sugar sensing in the early stages of Arabidopsis development. Loss-of-function mutants of RING-H2 E3 ligase *SUGAR-INSENSITIVE3* (*SIS3*) are hypersensitive to sucrose-mediated repression of cotyledon expansion and true leaves formation (Huang et al., 2010), but the molecular mechanism is still unclear since upstream regulators and SIS3 ubiquitination targets are still unknown. ATL15 is RING-H2 E3 ligase, which belongs to the family Arabidopsis Tóxicos en Levadura (ATL) that possess a characteristic N-terminal transmembrane domain that also regulates Arabidopsis development in response to sugars. The *ATL15* transcript is repressed by sugars, and the *atl15* knockout mutant is hypersensitive to high glucose concentrations during early seedling development (Aoyama et al., 2017). Starch levels are also lower in seedlings of *atl15* grown in high glucose concentrations, suggesting that *ATL15* could affect carbon partitioning. Further experiments under physiological conditions, i.e., older plants grown in soil and under equinoctial conditions, are needed to establish ATL15 as a regulator of carbon fluxes. Another ATL family member, ATL8, is induced by sugar starvation and interacts with STARCH SYNTHASE4 (SS4) in the split-ubiquitin assay (Luo et al., 2019). The results suggest that ATL8 could also be involved in starch metabolism. However, protein-protein interaction assays in plant cells are needed to demonstrate the co-localization of these proteins.

## Carbon Transport and Partitioning: The Sugar Flow From Source to Sink

Understanding the mechanisms by which plants allocate resources to different processes in a sink-source context is fundamental to improving and domesticating crops. For example, during seed development and filling, the plant destinates substantial amounts of carbon, nitrogen, and other essential elements to the seed. The resources stored in the seeds are required for the first stages of seedling development when photosynthesis and nutrient uptake are not an option yet. The nutrient-rich seeds are extensively used in human/animal feeding and in a variety of technological applications. Thus, seed quality is a major plant trait for crop improvement. To balance the relative amounts of starch and proteins in the seeds, plants constantly monitor the carbon/nitrogen ratio (C/N status), seeking its homeostasis.

**FIGURE 4:**
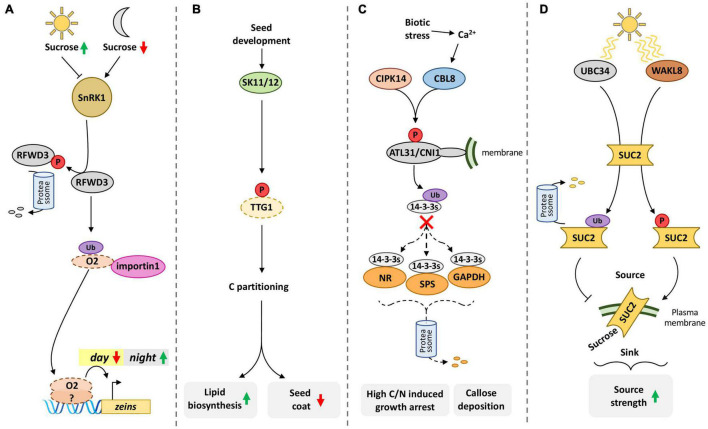
PTMs orchestrate the source-sink relationship. Integrating phosphorylation (red circles) and ubiquitination (purple ellipses) of specific enzymes and TFs is pivotal to controlling carbon fluxes throughout the plant. **(A)** SnRK1 integrates metabolic information to control carbon/nitrogen (C/N) balance in maize seeds. During the night, low sucrose availability in the endosperm activates SnRK1, which in turn phosphorylates the E3 ubiquitin ligase RFWD3 to trigger its ubiquitination and subsequent proteasomal degradation. Conversely, high sucrose availability during daytime inhibits SnRK1 and, consequently, stabilizes RFWD3. RFWD3 ubiquitinates the basic leucine zipper (bZIP) O2 transcription factor, promoting O2 interaction with importin1, the α-subunit of Importin-1, to enhance O2 nuclear localization and activate the expression of *zein* genes. The activation of *zein* genes enhances storage protein accumulation in maize seeds to balance their C/N status. **(B)** Phosphorylation of TTG1 by SK11/12 modulates TTG1 protein-protein interaction patterns, shifting seed metabolism between lipid biosynthesis and the production of mucilage pigments in the seed coat. **(C)** CIPK14 and CBL8 act synergistically to phosphorylate ATL31/CNI1, which in turn ubiquitinates 14-3-3s proteins. 14-3-3s degradation might destabilize SPSs, NR, and GAPDH, shifting C/N metabolism and possibly modulating growth according to C/N balance. This mechanism could also modulate carbon flow to biotic responses by adjusting callose deposition, a costly metabolic process, and a strong carbon sink. **(D)** The SUC2 sucrose transport activity is modulated by light intensity by the concerted action of UBC34 and WAKL8. While the E3 ubiquitin-conjugating enzyme UBC34 ubiquitinates and targets SUC2 for degradation under normal light, the phosphorylation of SUC2 by the wall-associated kinase WAKL8 enhances SUC2 activity in response to high light. SUC2 increased activity promotes sucrose transport from source to sink tissues to optimize growth under favorable conditions. This mechanism emphasizes how ubiquitination and phosphorylation integrate to adjust carbon homeostasis and source-sink relationships. Phosphorylation (P), ubiquitination (Ub). Connectors ending with arrows = activation; connectors ending with bars = repression. Solid connectors = demonstrated pathway; dashed connectors = hypothetic pathway. Green upward arrows = induction; red downward arrows = repression.

In this context, the central energy and carbon status sensor SnRK1 has emerged as a potential molecular target to optimize crop yield. Besides its fundamental role in adjusting plant metabolism to cope with low energy stress (LES), SnRK1 also controls the C/N balance, and thus starch/protein ratio, in maize seeds. A recent report showed that daily rhythms of sucrose concentration direct carbon and nitrogen accumulation in maize seeds through ZmSnRK1 (Li et al., 2020; Figure 4A). When sucrose levels are low, ZmSnRK1 phosphorylates the E3 ubiquitin ligase ZmRFWD3 and targets it for degradation. On the other hand, sufficient sucrose inhibits ZmSnRK1, allowing ZmRFWD3 protein accumulation. ZmRFWD3 ubiquitinates the bZIP transcription factor Opaque2 (O2), a major regulator of balanced C/N accumulation in maize seeds, increasing its nuclear localization during daytime by enhancing its interaction with importin1, the α-subunit of Importin-1 (Li et al., 2020). The cytonuclear distribution of O2 in maize endosperm follows a diurnal pattern that correlates with both sucrose and ZmRFWD3 levels, leading to enhanced transcription of zein genes during daytime. The loss-of-function *zmrfwd3* mutant has disrupted diurnal cytoplasmic O2 localization pattern and slight changes in seed C/N ratio due to decreased zeins and increased starch amounts. Li et al. (2020) work connects circadian rhythms of sugar status to the sink-source relationship and C/N ratio in maize seeds. The regulation of ZmSnRK1 by sucrose in maize seeds is likely to be mediated by T6P. Thus, SnRK1 may have a dual, but coordinated, function: manage cellular metabolism in response to cellular energy charge and modulate carbon and nitrogen fluxes throughout the plant. In Arabidopsis, the phosphorylation of TRANSPARENT TESTA GLABRA1 (TTG1) by SHAGGY-like kinases 11/12 (SK11/12) direct carbon flux to lipid biosynthesis and reduce the amount of carbon destinated to the seed coat, modulating the carbon partitioning between zygotic and maternal sinks (Li et al., 2018; Figure 4B).

The correct balance of C/N metabolism is essential to maximize plant fitness. The CBL-INTERACTING KINASE14 (CIPK14) acts together with CALCINEURIN B-LIKE8 (CBL8) to phosphorylate the RING-H2 ubiquitin ligase ARABIDOPSIS TOXICOS EN LEVADURA31/CARBON/NITROGEN INSENSITIVE1 (ATL31/CNI1) in a Ca^2+^-dependent manner (Yasuda et al., 2017). Transgenic Arabidopsis overexpressing ATL31/CNI1 is hyposensitive to the seedling growth arrest caused by high C/N ratio (Sato et al., 2009). The ATL31/CNI1 localization in membranes, possibly in the plasma membrane but not excluding other membrane organelles, is necessary for its role in C/N-mediated growth arrest. Phosphorylated ATL31/CNI1 binds to and ubiquitinates 14-3-3 proteins *in vitro*, suggesting that high C/N ratio induced ATL31/CNI1 phosphorylation is required for 14-3-3 repression under nutritional stress (Yasuda et al., 2017). The 14-3-3 proteins are known to regulate the stability of key enzymes involved in carbon and nitrogen metabolism. In sugar-starved Arabidopsis cells, the loss of 14-3-3 binding to SUCROSE PHOSPHATE SYNTHASE (SPS), NITRATE REDUCTASE (NR), and GLYCERALDEHYDE- 3-PHOSPHATE DEHYDROGENASE (GAPDH) is accompanied by the degradation of these critical metabolic enzymes (Cotelle, 2000; Figure 4C). Interestingly, the overexpression of ATL31 leads to accelerated callose deposition, a strong carbon sink, in response to powdery mildew penetration (Maekawa et al., 2014). Additionally, the tomato ATL31 ortholog might be involved in modulating starch degradation and the priming of callose deposition upon mycorrhizal inoculation (Sanmartín et al., 2021). One can hypothesize that the degradation of 14-3-3 proteins by ATL31 could lead to a metabolic shift of carbon utilization in response to biotic interactions.

The sucrose transporter SUC2 is a major proton-sucrose symporter responsible for loading the sucrose in the apoplast of source organs to the phloem for transport to sink tissues. The UBIQUITIN-CONJUGATING ENZYME 34 (UBC34) is an atypical E2-conjugating enzyme that ubiquitinates SUC2 in Arabidopsis, triggering SUC2 turnover in a light-dependent manner (Xu et al., 2020). In the same work, the authors also uncovered that SUC2 phosphorylation by the WALL-ASSOCIATED KINASE LIKE 8 (WAKL8) increases its activity in response to high light. The antagonistic relationship between UBC34 and WAKL8 in the regulation of SUC2 activity is highlighted by the phenotypes of the respective mutants: *ubc34* mutants show increased phloem loading and biomass accumulation while these parameters are reduced in *wakl8* (Figure 4D). Thus, ubiquitination and phosphorylation are dynamically integrated to adjust the activity of key proteins associated with carbon homeostasis.

## Carbon Utilization in Energy Metabolism: Glycolysis

The management of photoassimilates through interconversion of different sugars, sugar phosphates, and starch allows effective balancing of carbon and energy fluxes between tissues and organs during the diel cycle. The glucose breakdown through glycolysis generates ATP, NADH, and organic acid pyruvate. The pyruvate is further oxidized through the tricarboxylic acid (TCA) pathway in the mitochondria for further energy and reducing power production. In this section, we discuss the regulation of cytosolic glycolysis by PTMs.

The hexose-phosphorylating enzyme HXK1 possesses a glucose-sensing activity that is independent of its catalytic activity (Moore et al., 2003). HXK1 localizes in the nucleus upon glucose binding and mediates the repression of photosynthesis-related genes (Cho et al., 2006). Despite its central role in regulating carbon metabolism, we still have little knowledge of the regulation of HXK1 by PTMs. A recent report showed that the Arabidopsis HXK1 could be phosphorylated at S184 and S186 (Mergner et al., 2020). These phosphorylation sites are in the vicinity of the serine S177, which by homology can be the residue responsible for the phosphoryl transfer during catalysis. The substitution S177A in Arabidopsis renders a catalytically inactive HXK1 that still can sense glucose (Moore et al., 2003). Moreover, S177 corresponds to the S158 in the yeast Hxk2 (Feng et al., 2015), which was shown to facilitate phosphoryl transfer during glucose phosphorylation (Arora et al., 1991; Feng et al., 2015) and is necessary for catalysis (Heidrich et al., 1997). The kinetic analysis of HXK1 S184 and S186 phospho-mimic and phospho-null isoforms is necessary to establish putative roles of these residues phosphorylation in the HXK1 catalytic activity. Furthermore, previous *in vitro* proteome-wide screening seeking for SUMOylation targets identified the SUMOylation of Arabidopsis HXK1 (Elrouby and Coupland, 2010). This was later reinforced by phylogenetic analysis of HXK1, suggesting the existence of conserved SUMOylation motifs in plant hexokinases (Castro et al., 2020), and by HXK1 interaction with SUMO CONJUGATING ENZYME1 (SCE1) (Elrouby and Coupland, 2010). However, the *in vivo* occurrence and the possible physiological significance of HXK1 SUMOylation in plants remains to be investigated.

**FIGURE 5:**
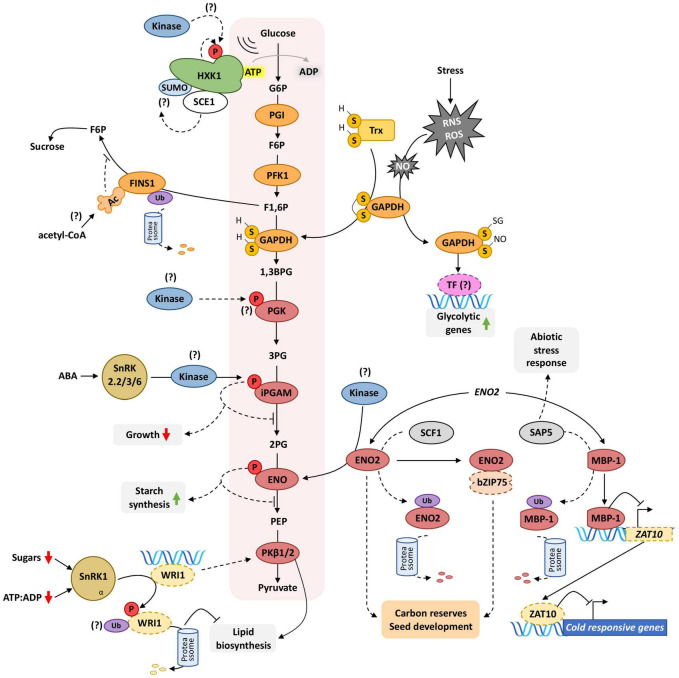
PTMs modulate carbon flow through glycolysis. Glycolysis is a highly conserved linear sugar catabolic pathway leading to the generation of ATP, NADPH, pyruvate, and metabolic intermediates for macromolecules biosynthesis. Thus, the elucidation of the PTMs targeting glycolytic enzymes is pivotal to understanding carbon and energy allocation in plants. HXK1, a hexose-phosphorylating enzyme mediating glucose entry in the glycolysis catabolic pathway, is phosphorylated near its active site, which could impact its glucose-sensing or catalytic activities. It is still unclear if the phosphorylation results from the activity of an upstream kinase targeting HXK1 or autophosphorylation. HXK1 also has a predicted SUMOylation site and interacts with the SUMO-conjugating enzyme SCE1, further supporting its putative SUMOylation. FINS1 redirect F1,6P from glycolysis to sucrose synthesis, possibly integrating metabolic information through acetyl-CoA mediated acetylation. FINS1 is ubiquitinated *in vivo* and possibly targeted for degradation to direct carbon toward glycolytic catabolism. The reduction of GAPDH mediated by reduced Trx increases its activity. Additionally, oxidative stress increases NO accumulation and triggers GAPDH S-glutathionylation and S-nitrosylation to promote the upregulation of glycolytic genes over GAPDH glycolytic activity. PGK is phosphorylated *in vivo*, but the upstream kinase and the physiological consequences of the phosphorylation are still unknown. The iPGAM phosphorylation relies on ABA signaling and SnRK2.2/3/6 action, which could promote growth arrest by diminishing glycolytic flux. The *ENO2* transcript can be translated to ENO2, which converts 2PG to PEP, or to MBP-1, lacking the ENO2 N-terminal region. MBP-1 inhibits the ZAT10 transcription to repress the ZAT10-mediated cold responses. SCF1 and SAP5 might ubiquitinate ENO2 and MBP-1, respectively, to control their accumulation. ENO2 interacts with bZIP75 to regulate carbon reserves during seed development. Here again, phosphorylation and ubiquitination seem to have a crucial role in regulating glycolysis to distribute C accordingly to metabolic and stress signals efficiently. The transcription factor WRI1, which modulates the transcription of PKβ1/2, is destabilized by SnRK1 direct phosphorylation, which may trigger the ubiquitination and degradation of WRI1 to regulate lipid biosynthesis. Ellipses and other rounded shapes = proteins (dashed lines: transcription factors (TFs), solid lines: enzymes). Phosphorylation (P), acetylation (Ac), sulfhydryl groups (S-H), disulfide bridge (S-S), ubiquitination (Ub), nitric oxide (NO), reactive oxygen species (ROS), reactive nitrogen species (RNS). Connectors ending with arrows = activation; connectors ending with bars = repression. Solid connectors = demonstrated pathways; dashed connectors = hypothetic pathways. Green upward arrows = induction; red downward arrows = repression. The consumption or the generation of ATP and NADPH by glycolysis were omitted for simplicity.

The next step of glycolysis involves the isomerization of G6P to fructose-6-phosphate (F6P) catalyzed by the moonlighting enzyme phosphoglucose isomerase (PGI). To this day, there are no reports of PTMs regulating PGI activity or subcellular localization in plants. Also, no PTMs of PHOSPHOFRUCTOKINASE1 (PFK1), the enzyme catalyzing the phosphorylation of F6P to produce fructose-1,6-bisphosphate (F1,6P), were identified to this date. The FRUCTOSE-1,6-BISPHOSPHATASE/FRUCTOSE INSENSITIVE 1 (FBPase/FINS1) dephosphorylates F1,6P, generating F6P for sucrose synthesis (Cho and Yoo, 2011). FINS1 was found to be ubiquitinated in a ubiquitinome assay using Arabidopsis cell-suspension cultures (Walton et al., 2016), suggesting that the ubiquitin-proteasome system (UPS) might regulate the FINS1 protein accumulation. FINS1 acetylation was also identified in another wide screening in Arabidopsis (Liu et al., 2018). One could hypothesize that FINS1 acetylation could potentially communicate the acetyl-CoA levels to reduce carbon flux through oxidative phosphorylation (Shi and Tu, 2015). Besides the role of FINS1 in modulating the distribution of fructose between respiration and sucrose synthesis, *FINS1* was also identified in a screening for fructose-insensitive mutants in Arabidopsis. The *fins1* mutant shows reduced photosynthetic rates, enhanced starch accumulation, and lower sucrose levels during the day (Rojas-González et al., 2015). Like HXK1, the FINS1 sugar-sensing ability is independent of its catalytic activity (Cho and Yoo, 2011; Figure 5).

Reactive oxygen species (ROS) and reactive nitrogen species (RNS) can regulate redox PTMs (e.g., carbonylation, glutathionylation, sulfhydryl oxidations, nitration, S-nitrosylation, and nitro-alkylation) and hold a crucial role during stress signaling (Turkan, 2018; Aranda-Caño et al., 2019; Ventimiglia and Mutus, 2020). The glycolytic enzyme GAPDH converts glyceraldehyde-3-phosphate (G3P) to 1,3-bisphosphoglycerate (1,3BPG), which can then be subsequently metabolized to 3-phosphoglycerate (3PG) and then to pyruvate for entering in the TCA cycle. Nitric oxide (NO) is a potent redox signaling molecule that leads to the formation of S-nitrosoglutathione and to protein S-nitrosylation. GAPDH, which might act as a NO sensor, is inactivated by S-glutathionylation and S-nitrosylation in the active site Cys159 (Hara et al., 2006; Holtgrefe et al., 2008; Schneider et al., 2018). GAPDH also interacts *in vivo* with thioredoxin-*h*3, which might revert GAPDH oxidative modifications. This might allow redox-dependent regulation of GAPDH to alter subcellular localization and subsequently its function, empowering it as a moonlighting protein. Moonlighting proteins show more than one physiologically relevant function (Jeffery, 2018). While oxidized GAPDH would locate in the nucleus to perform moonlighting functions (e.g., transcriptional activation of glycolytic genes during oxidative stress), Thx-mediated reduction of GAPDH would increase the GAPDH pool both in the cytosol and associate it with the mitochondria membrane to optimize the glycolytic flux (Figure 5; Schneider et al., 2018). Notably, an increasing number of glycolytic enzymes have been described to be S-glutathionylated, such as aldolase, A4-GAPDH, and cytosolic triosephosphate isomerase, which results in the latter’s inactivation (Figure 5; Ito et al., 2003; Michelet et al., 2005).

The PHOSPHOGLYCERATE KINASE (PGK) generates one ATP by transferring one phosphate from 1,3-bisphosphoglycerate (1,3BPG) to ADP, catalyzing the second step of the glycolysis sub-pathway that generates pyruvate from G3P. PGK is phosphorylated in the Ser201 (Van Leene et al., 2019), but the upstream kinase(s) and the stimulus that triggers the phosphorylation remain to be investigated. The next step of the pathway converting G6P to pyruvate involves the isomerization of 3-phosphoglycerate (3PG) to 2-phosphoglycerate (2PG) catalyzed by 2,3-BIPHOSPHOGLYCERATE-independent PHOSPHOGLYCERATE MUTASE (iPGAM). The Arabidopsis double mutant *ipgam1 ipgam2* has impaired stomatal movements in response to blue light, ABA, and low CO_2_ (Zhao and Assmann, 2011). Both vegetative and reproductive growths are severely impaired in *ipgam1 ipgam2*. Interestingly, ABA induces the phosphorylation of iPGAM1 Ser17 in an *SnRK2.2/2.3/2.6*-dependent manner (Wang et al., 2013). These findings suggest that iPGAM1 ABA-induced phosphorylation could repress its activity to repress growth.

The next step of glycolysis is converting 2PG to phosphoenolpyruvate (PEP) catalyzed by the enolase (ENO2 and ENO3 in Arabidopsis). Interestingly, the Arabidopsis *ENO2* locus encodes two alternative translation products starting at distinct translational start sites. The full-length protein (48 kDa) encodes the glycolytic enzyme enolase that catalyzes PEP production in the cytoplasm, while the alternative translation product MBP-1-like protein (AtMBP-1 = 37 kDa) localizes in the nucleus acting as a transcriptional regulator (Kang et al., 2013). AtMBP-1 represses the transcription of the zinc finger TF ZAT10 through direct binding to the promoter to repress cold response (Hojoung et al., 2002). AtMBP-1 also regulates the expression of ABA-signaling genes and is ubiquitinated by the E3 ubiquitin ligase STRESS-ASSOCIATED PROTEIN5 (SAP5), a positive regulator of abiotic stress responses (Kang et al., 2011, 2013). The Arabidopsis mutant *eno2* has altered starch and glucose levels in vegetative tissues and reduced seed size and weight. The reduced seed weight could be due to the impairment of cell proliferation, which could be caused by the lower cytokinin levels and/or the altered sugar status found in the mutant (Liu et al., 2020). In the same work, the authors found that ENO2 interacts with the bZIP75 transcription factor instead of AtMBP-1. ENO2 is phosphorylated at Ser56 (Roitinger et al., 2015) and Ser275 (Nakagami et al., 2010) in Arabidopsis, but the effects on the enolase activity and the physiological outcomes are still unknown. ENO2 also interacts with the SUMOylation enzyme SCE1, suggesting that it could also be SUMOylated (Elrouby and Coupland, 2010). In maize, the enolase ZmENO1 is phosphorylated in developing seeds in the eukaryotic conserved phosphorylation site Ser43. The phosphorylation is likely to modulate enzyme activity since the phosphomimetic isoform (Ser43Asp) shows decreased enolase activity *in vitro* (Cao H. et al., 2019). The phosphorylation of ZmENO1 positively correlates with starch accumulation in seeds (Cao H. et al., 2019).

The enzyme pyruvate kinase (PK) transfers a phosphate group from PEP to ADP, generating one pyruvate and one ATP. There are 14 loci encoding putative PKs in Arabidopsis; four of each are plastidial (Wulfert et al., 2020). Two plastidial PKs, namely PKβ1 and PKβ2, regulate the carbon flow to lipid biosynthesis in Arabidopsis seeds (Andre et al., 2007; Baud et al., 2007). Interestingly, the seed phenotype of *pk*β*1 pk*β*2* double mutant resembles the wrinkled phenotype of the WRINKLED1 mutant *wri1*, being the transcription of both PKβ1 and PKβ2 induced by WRI1 (Baud et al., 2007). Since SnRK1 negatively regulates the WRI1 stability through phosphorylation, the SnRK1-WRI1 pathway is likely conveying carbon status information to allocate carbon in Arabidopsis seeds correctly. SnRK1 seems critical for carbon partitioning in reproductive tissues of Angiosperms since maize ZmSnRK1 modulates the C/N status in seeds, as previously discussed (Li et al., 2020; Figure 4).

## Future Perspectives

Post-translational modifications greatly expand the plant proteome configurations by adding control layers beyond transcription-translation and allosteric regulatory mechanisms. The PTMs also allow dynamic and reversible changes in protein activity and subcellular localization, enabling plant adaptation to environmental changes in a resource-wise manner. New techniques and increasing computational power are now enabling high throughput and precise identification of PTMs.

However, there are still bottlenecks dampening the identification of the physiological significance of PTMs. The effects of PTMs on protein activity or localization must be analyzed through *in vitro* and reverse genetic approaches, usually one modification at a time. Additionally, the complexity of carbon metabolism in plants, which pervades many cellular organelles at the subcellular level and in long-distance transport and communication at the systemic level, makes it harder to get a comprehensive picture of the effects of PTMs on carbon fluxes. The development of high throughput gene-editing technologies coupled with rapid automated phenotyping platforms could solve this issue in a mid- to long-term timescale. PTM sites identified through *in vivo* large-scale proteomic analysis could guide gene editing to modify the modified target amino acid residue precisely, followed by an automated phenotypic screen of dozens, maybe hundreds, of lines. However, further optimization of gene editing methods for plants allowing the substitution of specific amino acids is required for large-scale screenings. Measurements of photosynthetic capacity or growth rates are already performed at large scales. Cheaper and automated sample processing and analysis are also quintessential to analyzing the effects of these PTMs on plant physiology. The wide array of metabolites (e.g., sugars, phosphate-sugars, and secondary metabolite intermediates) composing the path of carbon fluxes in the plant must be consistently and precisely measured, particularly those with low accumulation such as T6P.

The data collected in such high throughput proteomics-gene editing-phenotyping-“omics” pipelines using model plants, such as Arabidopsis and rice, can be fed into kinetics’ mathematical modeling (Nägele and Weckwerth, 2014) or machine learning algorithms. Machine learning can boost predictive models to identify both putative PTMs of new proteins and their physiological effects on the organism (Greener et al., 2022). Ensemble models using multiple machine learning algorithms might be deployed to analyze different parameters affecting plant metabolism. For example, deep learning algorithms (e.g., AlphaFold) could be used to predict changes in protein structure and enzyme activity induced by PTMs. The predicted changes in enzyme activity could be concatenated with machine learning models assessing the changes in metabolic fluxes, composing an ensemble model that could estimate the accumulation of reaction products. These trained models can be used to predict PTMs and their physiological consequences in less-studied species and crops with complex genome organization or long-life cycles, such as sugarcane and many trees, respectively. Mathematical modeling of the interaction between the circadian clock on sugar and starch metabolism exemplifies how computation can reveal clues on physiological outputs (Pokhilko et al., 2014; Webb and Satake, 2015). However, these models essentially consider the transcription-translational feedback regulations of the genes involved. Enhanced proteomic and metabolomic analysis capabilities will enable better models, which will speed up the discovery of new phenomena and improvement of crops in the climate change scenario.

## Author Contributions

All authors listed have made a substantial, direct, and intellectual contribution to the work, and approved it for publication.

## Conflict of Interest

The authors declare that the research was conducted in the absence of any commercial or financial relationships that could be construed as a potential conflict of interest.

## Publisher’s Note

All claims expressed in this article are solely those of the authors and do not necessarily represent those of their affiliated organizations, or those of the publisher, the editors and the reviewers. Any product that may be evaluated in this article, or claim that may be made by its manufacturer, is not guaranteed or endorsed by the publisher.

## Footnotes

1
https://www.psb.ugent.be/webtools/ptm-viewer
